# Attitudes Toward Artificial Intelligence, Career Engagement, and Intrinsic Motivation Among Healthcare Workers in Türkiye: A Cross-Sectional Study of Indirect Associations

**DOI:** 10.3390/healthcare14183078

**Published:** 2026-09-18

**Authors:** Burhanettin Uysal, Hilal Kamer, Abdurrahman Yunus Sarıyıldız, Ramazan Tiyek, Ömer Faruk Aslan

**Affiliations:** 1 Faculty of Health Sciences, Bilecik Şeyh Edebali University, Bilecik 11230, Türkiye; burhanettin.uysal@bilecik.edu.tr; 2 Independent Researcher, Istanbul 34870, Türkiye; 3 Faculty of Humanities and Social Sciences, Samsun University, Samsun 55080, Türkiye; yunus.sariyildiz@samsun.edu.tr; 4 Faculty of Economics and Administrative Sciences, Kırklareli University, Kırklareli 39000, Türkiye; ramazan.tiyek@klu.edu.tr; 5 Oltu Vocational School, Atatürk University, Erzurum 25400, Türkiye; ofaruk.aslan@atauni.edu.tr

**Keywords:** artificial intelligence, career engagement, intrinsic motivation, nursing management, digital transformation, healthcare workforce

## Abstract

**Highlights:**

**What are the main findings?**

- A statistically significant indirect association between attitudes toward artificial intelligence and intrinsic motivation was observed through career engagement among healthcare workers.
- Positive attitudes toward artificial intelligence were associated with higher career engagement and greater intrinsic motivation.

**What are the implications of the main findings?**

- Supporting career engagement may enhance healthcare workers’ motivation during the implementation of artificial intelligence in healthcare settings. Career engagement may be a relevant workforce factor in understanding healthcare workers’ motivational responses to AI-related change.
- Strategies that promote positive attitudes toward artificial intelligence may facilitate workforce adaptation to digital transformation in healthcare. Longitudinal and intervention studies are needed to determine whether career-focused or AI-related strategies influence motivation or adaptation.

**Abstract:**

**Background/Objectives**: Artificial intelligence (AI) is increasingly reshaping healthcare by influencing professional roles, learning demands, and career development. Guided by Self-Determination Theory and Social Cognitive Career Theory, this study examined the indirect association between attitudes toward AI and intrinsic motivation through career engagement among healthcare workers while accounting for demographic and occupational characteristics. **Methods**: A cross-sectional online survey was conducted among 902 healthcare workers in Türkiye. Ordinal confirmatory factor analyses based on polychoric correlations were estimated using diagonally weighted least squares. Alternative measurement models and one theoretically justified wording-method association were evaluated. Covariate-adjusted regression mediation analyses were performed using HC3 robust standard errors and 5000 nonparametric bootstrap resamples with bias-corrected confidence intervals. Common method variance, effect sizes, predictive relevance, subgroup analyses among nurses and midwives, and a sensitivity analysis using a 20-item intrinsic motivation scale were also examined. **Results**: The selected measurement models demonstrated acceptable to excellent fit, although the GAAIS S-1 model showed borderline RMSEA and two reverse-worded IMSE items retained weak loadings. A statistically significant indirect association between attitudes toward AI and intrinsic motivation was observed through career engagement (indirect association = 0.099, 95% bias-corrected bootstrap CI [0.071, 0.132]), accounting for 27.9% of the total association. The 20-item sensitivity analysis yielded a similar indirect association (indirect association = 0.105, 95% bias-corrected bootstrap CI [0.075, 0.139]). Profession-stratified analyses showed significant indirect associations in both occupational strata, although the AI-attitude–career-engagement association was stronger among nurses/midwives. **Conclusions**: More favorable attitudes toward AI were associated with greater career engagement and intrinsic motivation. Career engagement explained a meaningful, although not dominant, proportion of this association. Because the data were cross-sectional and self-reported, the findings should be interpreted as associative rather than causal. Supporting career engagement may be a plausible target for future intervention research aimed at facilitating healthcare workers’ adaptation to AI-enabled environments, although adaptation itself was not directly assessed in this study.

## 1. Introduction

Artificial intelligence (AI) refers to computational systems capable of performing tasks that typically require human cognitive functions such as learning, prediction, and decision-making. In healthcare, AI is increasingly embedded in clinical, administrative, and patient-centered processes, making it a central component of ongoing health system transformation. According to this definition, AI is considered not merely a technology or product, but also a social and cognitive phenomenon. In summary, AI is the automation of cognition [1]. The term AI is a general term that can be used to describe any computer software engaged in tasks similar to those performed by humans, such as learning, planning, and problem solving [2]. 

As in many other fields, AI has begun to be widely used in the healthcare sector. The main application categories include diagnosis and treatment recommendations, patient engagement and adherence, and administrative activities. The complexity and growth of data in healthcare services means that AI will be applied increasingly in this field [3,4]. Through technologies that can predict, comprehend, learn, and act (ranging from identifying new relationships between genetic codes to controlling robots that assist in surgeries) AI is revolutionizing and empowering modern healthcare services [5]. Despite the growing adoption of AI in healthcare, limited research has examined its implications for workforce motivation and health system performance simultaneously.

There is great optimism that the implementation of AI can bring significant improvements across all areas of healthcare, from diagnosis to treatment. It is generally thought that AI tools will facilitate and enhance human work but will not completely eliminate the jobs of doctors and other healthcare personnel. While AI is poised to support healthcare staff in various tasks ranging from administrative workflows to clinical documentation and patient information, it can also offer specialized support such as image analysis, medical device automation, and patient monitoring [6]. AI, whose presence today is considered inevitable, currently focuses on recognizing objects using sensors and then performing the appropriate action according to pre-programmed rules. It still has a limited level of impact. Nevertheless, concerns related to AI (such as data privacy, social issues, ethical issues, cyberattack issues, and developer issues) remain as obstacles to the successful implementation of AI in the medical sector [7].

Motivation can be described as the latent force that initiates, directs, activates, sustains, and ultimately terminates the behaviors of individuals or organisms. It occupies an important place as a concept in human success. For example, wanting to succeed in an exam and thinking about studying for it are actions related to motivation [8]. Motivation is defined as a psychological process that enables the initiation and maintenance of goal-directed behaviors [9]. 

The motivation of healthcare workers is an important concept in achieving efficient, effective, and high-quality healthcare delivery. Since healthcare delivery is extremely labor-intensive, human resources are recognized as critically important in the efforts of healthcare organizations to achieve their organizational goals. Although motivation has a cultural dimension, compensation, managerial support, and career advancement are key factors affecting all healthcare professionals regardless of country [10]. In addition, the motivation of healthcare workers is influenced by factors related to supervision, financial benefits, job training, and growth [11]. The results of one study revealed that among four main motivational factors, achievements ranked first, followed by compensation, colleagues, and job characteristics, respectively. Healthcare workers tend to be motivated more by intrinsic factors, which means this should be a target for effective employee motivation [12]. Intrinsic motivation is affected by many conditions, one of which is the sense of trust. Research shows that trust relationships in the workplace encourage social interactions and cooperation among healthcare workers, affect the intrinsic motivation of healthcare workers, and produce outcomes in terms of work attendance, performance, and quality of care [13]. Significant productivity increases occur in employees with high intrinsic motivation. Indeed, the findings of a study conducted in this context showed that the most important intrinsic motivation factors provided up to an 80% increase in productivity in organizations [14]. Another study demonstrated that staff motivation in the healthcare sector has an effect on employee performance and job satisfaction, and that there is a strong relationship between motivation, employee performance, and job satisfaction [15]. Along with intrinsic motivation, employees’ motivation and therefore productivity also increase when they feel they are treated equally, make decisions collectively, are respected, can be autonomous, and are recognized as valuable [16].

Another topic that needs to be evaluated within the scope of the research is career engagement. Engagement is a force that binds an individual to a course of action related to one or more goals. Therefore, engagement can be distinguished from change-based forms of motivation and goal-related attitudes and can influence behavior even in the absence of extrinsic motivation or positive attitudes [17]. Operationally, career engagement refers to an individual’s degree of proactive behaviors aimed at managing and advancing their career development, such as career planning, networking, and skill enhancement [18]. It is critical to distinguish career engagement from related attitudinal constructs: unlike organizational commitment (which binds an individual to a specific institution) or career commitment (which reflects an affective attachment or attitude toward a profession), career engagement specifically captures actionable, proactive career behaviors [18,19]. Individuals with high career engagement tend to set career goals and make efforts to achieve them [19]. 

In a study on the career engagement of nurses who are among the healthcare sector employees a positive and significant correlation was found between nurses’ perceptions of their profession and their engagement with their nursing career [20]. Perception related to the profession directly affects career engagement. Results obtained from another study found that employees with high career engagement had moderate levels of job satisfaction and organizational engagement, and a weaker intention to leave [21]. Job satisfaction, perceived job security, and career satisfaction directly affect employees’ career engagement. This is important in terms of the continuity of employment of qualified workers [22].

Although the literature includes various studies on artificial intelligence applications in the healthcare sector, research specifically examining the mediating role of career engagement in the relationship between AI adoption and employee motivation remains quite limited. Nevertheless, it is understood that numerous studies have been conducted in different sectors on AI, employee motivation, and career engagement. One of these is the study in which transformational leadership and the perception of AI use were found to play separate mediating roles in the relationship between nurses’ happiness and innovative work behavior. The related study focused on the effect of transformational leadership and AI applications on nurses’ happiness [23]. Another study yielded results that would contribute to achieving outcomes encouraging the adoption of AI-supported health technologies. It demonstrates that job resources, job demands, and employee engagement serially mediate the relationship between resistance to AI-supported health technologies and behavioral intentions to adopt AI [24]. In a recent study conducted in the Turkish context, a cross-sectional study involving data collected from 388 nurses found that nurses’ level of knowledge about artificial intelligence was limited; however, they held an optimistic attitude regarding work efficiency, quality of care, and the reduction in burnout. In contrast, patient privacy and ethical concerns emerged as the primary barriers to integration [25].

Consistent with these findings in the Turkish context, a methodologically focused study by Öztürk Yıldırım and Karaman (2025) [26] developed a four-factor “Artificial Intelligence Attitude Scale for Nurses” with high validity and reliability, designed to assess Turkish nurses’ attitudes toward artificial intelligence. This scale-development study demonstrates the strategic importance of measuring clinical staff’s attitudes and perceptions with appropriate instruments for the success of digital transformation in healthcare [26].

In another study conducted with 288 nurses across Turkey, participants’ general attitudes toward artificial intelligence and generative tools such as ChatGPT were found to be positive (GAAIS: 67.54 ± 13.14). Of the participants, 48.3% reported being aware of these tools, while 27.8% reported actively using them; those who used the technology were found to have higher levels of positive attitudes. Furthermore, 84.4% of nurses argued that they should be informed on this subject, while 58.3% argued that these tools would contribute to patient care [27].

Similarly, a study conducted with 737 nurses across three different provinces in eastern Turkey found that attitudes toward artificial intelligence were highly positive. The analysis results showed that demographic and institutional/regional factors were determinants of these attitudes (*p* < 0.05). When all these local findings are evaluated holistically, it can be understood that nurses in Turkey are open to artificial intelligence integration, but that factors such as knowledge gaps, regional differences, and ethical concerns directly affect this process [28].

Building upon these empirical insights regarding workforce perception and technological transformation, in another study investigating the effect of AI on employees’ work, it was concluded that AI applications in diagnosis and treatment, patient engagement and empowerment, and administrative activities have an effect on various components of healthcare workers’ job design [29]. While AI offers opportunities to reduce administrative burden, promote teamwork, and improve patient care, it also presents challenges related to technology adaptation and employees’ concerns about automation. The findings of another study, in which the impact of AI adoption on hospital staff’s job satisfaction and productivity was assessed using a qualitative methodology, reveal that AI enhances job satisfaction by simplifying documentation, supporting skill development, improving decision-making processes, and creating innovation opportunities [30]. In yet another study, it was concluded that the adoption of AI in Human Resources Management positively affects employee motivation and performance. The study emphasizes the importance of AI transparency, user-friendliness, and ethical implementation in creating a motivated and high-performing workforce [31]. This interplay between AI use and motivational dynamics is further supported by the integrative review conducted by Shinners et al. (2020) [32]. The authors found that intrinsic motivation plays a fundamental role in healthcare workers’ adoption of AI-supported clinical decision support systems, and that employees’ motivation and willingness to use AI declined markedly when the technology’s benefit to patients was not clearly understood or when trust in the technology was insufficient [32].

This study aims to bring a holistic perspective to the existing literature by examining the relationship between AI applications and the intrinsic motivation of healthcare workers through the potential mediating role of career engagement. Although the increasingly widespread use of AI technologies in healthcare services today is transforming the ways in which employees work and their sources of motivation, studies on how this transformation is shaped through individuals’ engagement with their careers appear to be limited. In particular, addressing intrinsic motivation not only in the context of technological innovations, but also in relation to the individual’s professional belonging and long-term career goals points to an important gap in the literature. In this context, the study aims to shed light on the relationship between technological transformation and individual professional attitudes, and to address this deficiency in the field, by theoretically proposing and examining the association between AI attitudes and the motivation of healthcare workers through the potential mediating role of career engagement.

Beyond its individual-level contributions, this study offers important implications for health system planning and management. Examining how attitudes toward AI are associated with healthcare workers’ career engagement and intrinsic motivation may assist policymakers and healthcare administrators in developing more effective workforce strategies, improving service delivery efficiency, and enhancing overall system performance. By emphasizing the human and motivational dimensions of AI adoption, the study contributes to social science literature by conceptualizing AI integration in healthcare as a socio-technical, workforce-centered process. In doing so, it extends existing technology adoption frameworks beyond purely cognitive and behavioral perspectives, highlighting the role of professional engagement in shaping responses to digital transformation.

### Research Hypotheses

Social cognitive career theory (SCCT), developed by Lent and colleagues (1994) building on Albert Bandura’s social cognitive theory, posits that an individual’s career trajectory emerges from the dynamic interaction of internal/subjective factors, external environmental factors, and behaviour [33,34]. In the healthcare sector, where technological transformation is accelerating, employees’ attitudes toward artificial intelligence are shaped as a reflection of cognitive appraisal processes and outcome expectations. Specifically, within the SCCT framework, positive or negative attitudes toward AI serve as cognitive proxies for outcome expectations—reflecting healthcare workers’ beliefs about the anticipated professional outcomes, efficiency gains, or disruptions resulting from technological integration [34]. Self-Determination Theory (SDT), which complements these cognitive and environmental dynamics, emphasizes that individuals possess an innate potential for growth, but that this potential is shaped through a dialectical interaction with environmental conditions [35]. Unlike traditional approaches, SDT focuses not on the amount of motivation but on autonomous and controlled forms of motivation and their effects on engagement, performance, and psychological well-being [36]. In SDT, intrinsic motivation is sustained through the satisfaction of three basic psychological needs: autonomy, competence, and relatedness [37]. It is crucial to clarify a conceptual distinction in our research design: while these three basic needs provide the theoretical rationale for why career engagement translates into intrinsic motivation, they function solely as explanatory theoretical constructs in our conceptual narrative and are not directly measured as empirical variables in the structural model [38].

The proposed directionality among the variables in the model (AI Attitude → Career Engagement → Intrinsic Motivation) rests on an analytical synthesis of SCCT and SDT principles. To rigorously establish this conceptual chain, each hypothesized association is detailed based on distinct underlying mechanisms while making a clear distinction between theoretical explanations and the actual tested processes:

- Direct Association between AI Attitude and Career Engagement (H1): According to SCCT’s choice and performance models [39], cognitive attitudes (acting as proxies for outcome expectations) toward technological environmental changes are theorized to align with goal-directed career behaviors. When healthcare workers perceive AI positively—expecting it to streamline administrative burdens rather than threaten their professional role—they display higher optimism about their future career prospects, which is hypothesized to relate to more proactive career behaviors and engagement, such as active career planning and skill adaptation.
- Direct Association between Career Engagement and Intrinsic Motivation (H2): From an SDT perspective [40], active career engagement serves as an action domain through which individuals experience professional growth. Through proactive career actions (e.g., self-directed learning and professional development), highly engaged employees are more likely to experience feelings of autonomy and competence in their professional role. Although need satisfaction itself is not empirically measured in this study, this theoretical mechanism explains why career-engaged individuals are expected to derive greater inherent joy, satisfaction, and intrinsic motivation from their work activities.
- Direct Association between AI Attitude and Intrinsic Motivation (H3): Beyond career engagement, favorable cognitive perceptions of technology may also be associated with a more positive work experience. Viewing AI tools as supportive rather than burdensome may coincide with lower perceived cognitive strain and greater perceived task efficacy, which are theoretically consistent with higher intrinsic motivation toward day-to-day work tasks.
- Hypothesized Indirect Association (H4): While digitalization has the potential to alter job autonomy and introduce psychosocial risks [41], positive AI attitudes are posited to mitigate risk perceptions and support proactive career planning and self-development. These proactive career behaviors, in turn, are hypothesized to provide the psychological foundation for intrinsic motivation. Accordingly, career engagement was examined as an intervening variable in a theory-informed indirect-association model linking AI attitudes with intrinsic motivation. Because the data were cross-sectional, this specification does not establish a temporal or causal mediating mechanism.

In the literature, the effects of digitalization and AI on healthcare workers have often been addressed only at the level of technology acceptance or general job satisfaction, and the mechanisms explaining how cognitive AI attitudes are translated into deeper psychological outcomes such as intrinsic motivation through career engagement, on the basis of SCCT and SDT, have been neglected. Building on this, the following hypotheses have been developed to test these theoretical claims among healthcare workers:

**Hypothesis** **1** **(H1).** *Attitudes toward artificial intelligence are positively associated with career engagement among healthcare workers*.

**Hypothesis** **2** **(H2).** *Career engagement is positively associated with intrinsic motivation among healthcare workers*.

**Hypothesis** **3** **(H3).** *Attitudes toward artificial intelligence are positively associated with intrinsic motivation among healthcare workers*.

**Hypothesis** **4** **(H4).** *Attitudes toward artificial intelligence are indirectly associated with intrinsic motivation through career engagement among healthcare workers*.

By focusing on intrinsic motivation and career engagement, this study contributes to applied health psychology by explaining how healthcare workers psychologically adapt to AI-driven changes in their work environment.

## 2. Methods

### 2.1. Research Design and Sample

This quantitative study used a cross-sectional online survey and was reported in accordance with the Strengthening the Reporting of Observational Studies in Epidemiology (STROBE) recommendations for cross-sectional studies. The study population comprised adults actively engaged in healthcare work or supervised clinical/professional practice within public or private healthcare settings in Türkiye. The sample also included 62 adult students/interns who were actively engaged in supervised clinical or professional practice within healthcare settings during the data-collection period. Although these participants were at a practice-based training stage rather than being classified within the established professional groups, they were directly involved in healthcare environments and were therefore retained as a distinct occupational category. Their inclusion was considered relevant to the study because they were actively exposed to the professional and technological environment in which AI-related changes in healthcare occur. The wording of the target population in the original manuscript was narrower than the operational composition of the recruited sample. The 62 students/interns were already included in the original dataset as a distinct occupational category and were adults undertaking supervised clinical or professional practice in healthcare settings; they were not added or reclassified after data collection. Accordingly, the revised eligibility description is intended to clarify the population represented in the original recruitment and analytic dataset rather than to retrospectively expand the study population. Data were collected voluntarily from April 2025 to February 2026 through a Google Forms questionnaire. Individual response dates or survey months were not retained in the analytic dataset; therefore, calendar time could not be included as a covariate or examined in a temporal sensitivity analysis.

Because institution-level access to healthcare workers was limited, participants were recruited through snowball sampling. Initial contacts were asked to circulate the survey within their professional networks. This approach facilitated access to geographically and professionally diverse respondents but did not provide a known sampling frame, calculable inclusion probabilities, or a formal response rate; therefore, the sample should be regarded as a non-probability convenience network sample rather than a nationally representative sample of healthcare workers in Türkiye. The study records indicated 1284 submitted questionnaires. Before statistical analysis, all submissions underwent questionnaire-level data-quality screening. Records were excluded if they were incomplete; contained clearly erroneous or internally inconsistent responses indicating incorrect completion; or displayed obvious non-differentiated response patterns suggestive of inattentive or non-serious responding, such as repeatedly selecting the same response option across a large number of items (straight-lining; e.g., consistently marking only 1 s or only 5 s). No participant was excluded on the basis of the substantive magnitude of the GAAIS, IMSE, or CES score, and no minimum or maximum construct-score threshold was used as an exclusion criterion. In total, 382 submissions were excluded during this screening process, leaving 902 records for analysis. Because the archived screening records did not retain reason-specific counts for each exclusion category, the number of exclusions attributable to each individual criterion cannot be reported retrospectively. The conventional minimum target sample was 384.

The sample included multiple occupational groups because AI implementation in healthcare is interprofessional and nursing work is embedded in shared clinical and administrative systems. Because nursing personnel constitute a large and clinically central subgroup of the healthcare workforce, and because AI implementation may affect professional groups to varying degrees, profession was included as a covariate in the primary models, and the mediation analysis was repeated separately for nurses/midwives and other occupational groups. Nevertheless, profession was treated as an adjustment variable rather than as a complete proxy for occupational context. The included occupational groups may differ in their degree of AI exposure, professional autonomy, career structures, and motivational conditions; therefore, residual occupational heterogeneity may remain even after statistical adjustment for profession.

### 2.2. Ethical Considerations 

This study was approved by the Samsun University Ethics Committee (Decision No. 2025/31) following review at the committee meeting held on 21 March 2025. Participation was voluntary. The survey introduction explained the purpose of the study, confidentiality arrangements, the informed consent procedure, and participants’ right to discontinue their participation at any time before submitting the questionnaire. Completion of the questionnaire was regarded as providing informed consent. No directly identifying personal information was collected, and all data were analyzed anonymously. The study was conducted in accordance with the ethical principles of the Declaration of Helsinki.

### 2.3. Data Collection Instruments

Intrinsic Motivation Scale for Employees (IMSE). The 22-item scale developed by [42] comprises five dimensions: Utility (items 1–6), Belief (items 7–10), Perceived Competence (items 11–15), Negative Affect (items 16–19), and Interest/Enjoyment (items 20–22). Items are rated on a 5-point Likert scale. A scoring audit showed that the supplied variables were already stored in the scored direction: the archived total equaled the item sum, and reversing items 10 and 12 a second time produced negative corrected item-total relationships. Therefore, no second reversal was applied. Higher scores indicate stronger intrinsic motivation.

Career Engagement Scale (CES). The 9-item instrument adapted into Turkish by [37] comprises proactive career planning/evaluation (items 1–5) and proactive career skill-development behavior (items 6–9). Items are rated on a 5-point Likert scale, with higher scores indicating stronger career engagement.

General Attitude Towards Artificial Intelligence Scale (GAAIS). The Turkish adaptation reported by [43] includes 12 positively worded and eight negatively worded items. Negative-attitude items were reverse-scored so that higher total scores consistently represented more favorable attitudes toward AI. Items are rated on a 5-point Likert scale.

### 2.4. Data Analysis

Statistical analyses were conducted using custom Python scripts developed for the present study. The primary computational environment was Python 3.13.5, with pandas 2.2.3, NumPy 2.3.5, SciPy 1.17.0, statsmodels 0.14.6, and scikit-learn 1.8.0 used for data management, numerical computation, regression-based analyses, and predictive validation procedures. The dataset was parsed and verified prior to analysis. The analytic workflow included data screening, verification of item scoring and reverse coding, reliability analysis, estimation of polychoric correlation matrices, ordinal confirmatory factor analysis using DWLS, construct-validity analyses (composite reliability [CR], average variance extracted [AVE], heterotrait–monotrait ratio [HTMT], and the Fornell–Larcker criterion), evaluation of alternative measurement models, covariate-adjusted regression mediation with HC3 heteroskedasticity-consistent standard errors, and bootstrap estimation of indirect associations. Descriptive statistics and preliminary data-management procedures were independently checked using JASP (Version 0.95.3; JASP Team, Amsterdam, The Netherland, 2025). Analytic scripts were organized so that the main data-processing, measurement-model, mediation, sensitivity, and subgroup analyses could be rerun from the prepared analytic dataset.

Because all substantive items used five ordered response categories, ordinal confirmatory factor analysis was estimated from polychoric correlation matrices using diagonally weighted least squares (DWLS) [44,45]. For each scale, the prespecified first-order structure was compared with plausible alternatives, including one-factor, correlated-factor, higher-order, bifactor, and S-1 bifactor specifications where identifiable. Model evaluation considered the DWLS chi-square statistic, degrees of freedom, Comparative Fit Index (CFI), Tucker–Lewis Index (TLI), RMSEA with its 90% confidence interval, Standardized Root Mean Square Residual (SRMR), and the Weighted Root Mean Square Residual (WRMR). Because DWLS is not likelihood-based, AIC and BIC were not calculated. Model fit was evaluated using multiple complementary fit indices rather than relying on a single cutoff criterion [46].

Modification indices and residuals were used diagnostically rather than as automatic model-building criteria. The largest theoretically interpretable IMSE modification involved the residual covariance between items 10 and 12, the scale’s only reverse-worded items; this single wording-method association was therefore estimated. No cross-loading or residual covariance was added solely to improve model fit. The published 22-item IMSE composition was retained as the primary operationalization to preserve fidelity to the original instrument and to avoid making post hoc, sample-informed item deletion the basis of the primary hypothesis test. Because items 10 and 12 showed weak loadings in the present data, a 20-item specification excluding these items was examined as a sensitivity analysis. Although the 20-item specification showed improved psychometric fit, it was not treated as a newly validated replacement for the published 22-item instrument because the item removal was data-informed and evaluated within the same sample. The purpose of the 20-item analysis was therefore to test whether the substantive indirect association depended on retention of the two weak items rather than to redefine the IMSE scale. Convergent and discriminant validity were evaluated using standardized loadings, composite reliability (CR), average variance extracted (AVE), factor correlations, the Fornell–Larcker criterion, HTMT, and, for bifactor representations, explained common variance (ECV), omega hierarchical (ωH), omega total, and percentage of uncontaminated correlations.

Common method variance was screened using Harman’s unrotated single-factor test across all 51 substantive items [47]. This procedure was treated only as a diagnostic screen and not as a statistical test capable of establishing the absence of common method bias. Its result was therefore interpreted in conjunction with the same-source, same-time self-report design.

The latent structural model was initially specified as the intended primary framework for hypothesis testing. The initially specified latent structural model was defined at the subscale-composite level using nine observed indicators. The latent AI-attitude factor was indicated by the positive-attitude (PGAAIS) and negative-attitude (NGAAIS) GAAIS subscale scores; career engagement was indicated by the planning/evaluation (PLEV) and research/networking (RENE) subscale scores; and intrinsic motivation was indicated by the Utility, Belief, Perceived Competence, Negative Affect, and Interest/Enjoyment IMSE subscale scores. The structural component specified paths from AI attitudes to career engagement, from career engagement to intrinsic motivation, and directly from AI attitudes to intrinsic motivation. Because these indicators were continuous subscale composite scores, the latent structural model was estimated by maximum likelihood (ML) from the covariance matrix of the nine subscale indicators, whereas the item-level ordinal measurement models described above were estimated using DWLS. For scale identification, one loading per latent factor was fixed to 1.0 (PGAAIS for AI attitudes, PLEV for career engagement, and Utility for intrinsic motivation), while the remaining six primary factor loadings were freely estimated. All nine indicator residual variances were freely estimated; cross-loadings and correlated indicator residuals were fixed to zero. The variance of the exogenous AI-attitude factor and the residual variances of the two endogenous latent factors were freely estimated, and no residual covariance was specified between the endogenous latent factors. With nine observed indicators, the covariance matrix contained 45 unique sample moments. The model estimated 21 free parameters—six factor loadings, nine indicator residual variances, three structural regression paths, and three latent variance/disturbance parameters—yielding 24 degrees of freedom (45 − 21 = 24). The full specification of the rejected latent structural model is provided in Supplementary Table S5. However, after examination of its global model fit, the model was not retained for primary inference because its overall fit was inadequate. Thus, the decision to transition from latent-variable modeling to observed-score mediation was made after evaluation of the latent structural model and should be regarded as an analysis-stage, post-fit modification rather than a prespecified primary strategy. The observed-score approach was selected because the substantive hypotheses concerned associations among the overall AI-attitude, career-engagement, and intrinsic-motivation constructs, while the measurement analyses provided stronger support for interpretation of overall scores than for fine-grained latent subdimensions. For the GAAIS, general-factor evidence supported overall-score interpretation. For the IMSE, the use of the exact 22-item composite employed in the main post-fit observed-score analysis was supported by its high total-score internal consistency (Cronbach’s α = 0.971), the acceptable fit of the 22-item five-factor measurement model including the single theoretically justified reverse-wording residual association (CFI = 0.998, TLI = 0.998, RMSEA = 0.069, SRMR = 0.049), and adequate composite reliability and average variance extracted across the five first-order dimensions. However, these findings were not interpreted as evidence of strict unidimensionality because two reverse-worded items performed weakly and discriminant validity among several IMSE dimensions was limited. Importantly, the stronger general-factor evidence obtained for the modified 20-item bifactor model was treated only as sensitivity evidence and was not used to establish the validity of the original 22-item composite. Accordingly, standardized total scores were used in a covariate-adjusted regression mediation framework rather than interpreting structural paths from a poorly fitting latent model. This analytical transition has important methodological implications. Unlike latent-variable models, observed-score regression does not explicitly separate true-score variance from measurement error. Consequently, unreliability in the composite scores may attenuate associations, and other forms of measurement error may introduce additional bias into the estimated regression and indirect-association coefficients. Standardization, HC3 robust standard errors, and bootstrap confidence intervals address scale comparability, heteroskedasticity, and sampling uncertainty, respectively, but they do not correct for measurement error. The resulting coefficients should therefore be interpreted as associations among observed composite scores rather than as measurement-error-corrected latent effects.

All focal total scores were standardized. Age, gender, marital status, education, profession, work unit, work schedule, and weekly working hours were entered as controls. Covariates were entered using their original analytic coding. Age was treated as an ordered covariate (1 = ≤25 years, 2 = 26–35 years, 3 = 36–45 years, and 4 = ≥46 years). Gender was coded 1 = women and 2 = men, and marital status was coded 1 = married and 2 = single; thus, the corresponding coefficients represent men relative to women and single participants relative to married participants. Education was treated as an ordered covariate (2 = secondary education, 3 = associate degree, 4 = bachelor’s degree, 5 = master’s degree, and 6 = doctorate). Profession was dummy-coded with physicians as the reference category (1 = physicians, 2 = nurses/midwives, 3 = health technicians, 4 = other health professionals, 5 = managers/specialists, 6 = administrative/support personnel, and 7 = students/interns). Work unit was dummy-coded with clinical and inpatient wards as the reference category (1 = clinical and inpatient wards, 2 = emergency and intensive care units, 3 = operating room/delivery room/sterilization, 4 = administrative and management units, 5 = diagnostic/support/technical units, 6 = patient services and primary care, and 7 = education and other). Work schedule was dummy-coded with on-call work as the reference category (1 = on-call, 2 = day, 3 = shift, and 4 = mixed). Weekly working hours were treated as an ordered covariate (1 = <40 h, 2 = 40 h, and 3 = >40 h). Accordingly, age, education, and weekly working-hours coefficients represent a one-category increase, whereas coefficients for dummy-coded covariates represent contrasts with their stated reference categories. HC3 heteroskedasticity-consistent standard errors (HC3) were used for regression paths. The indirect association was evaluated using 5000 nonparametric bootstrap resamples, with a 95% bias-corrected (BC) bootstrap confidence interval [48]. Mediation magnitude was summarized using variance accounted for (VAF); R^2^, adjusted R^2^, Cohen’s f^2^, and Stone–Geisser predictive relevance (Q^2^) were also reported. The main post-fit observed-score analysis used the 22-item IMSE score. Sensitivity analyses used the 20-item IMSE score. Profession-stratified analyses repeated the post-fit observed-score model separately for nurses/midwives (n = 266) and all other occupational groups (n = 636), using the same standardized focal scores and covariate structure as in the main post-fit observed-score analysis. Profession was omitted from the nurses/midwives subgroup model because it was invariant within that subgroup but was retained as a categorical covariate in the other-occupational-groups model. Between-group heterogeneity was formally examined by adding nurses/midwives-by-predictor interaction terms to the corresponding regression models. To assess whether inclusion of students/interns influenced the primary findings, an additional sensitivity analysis repeated the full covariate-adjusted model after excluding all 62 participants classified as students/interns, yielding a restricted analytic sample of 840 participants. The focal scores were restandardized within this restricted sample, and the same covariate structure, HC3 heteroskedasticity-consistent standard errors, and 5000-resample bias-corrected bootstrap procedure used in the main post-fit observed-score analysis were retained; the students/interns profession category was necessarily absent from this restricted model. Two-sided *p* values below 0.05 were considered statistically significant.

## 3. Results

### 3.1. Participant Characteristics

The sample comprised 902 healthcare workers. Participants aged 25 years or younger constituted 42.0% of the sample, 66.4% were women, and 62.2% held a bachelor’s degree. Nurses and midwives were the largest professional group (29.5%), followed by physicians (20.1%) and health technicians (18.3%). Clinical and inpatient wards constituted the largest work-unit category (31.9%), followed by education and other units (15.0%) and diagnostic, support, and technical units (14.4%). Slightly more than half worked a day schedule (51.2%), and 80.0% reported working 40 h or more per week (Table 1).

### 3.2. Descriptive Statistics and Reliability

Mean scores indicated moderately favorable attitudes toward AI, relatively strong career engagement, and high intrinsic motivation. Internal consistency was high for all three total scores (α = 0.936–0.971). Bivariate correlations among the total scores were positive and statistically significant (Table 2).

### 3.3. Ordinal Measurement Models

The ordinal analyses improved model evaluation but did not justify claiming perfect measurement. For GAAIS, the correlated two-factor specification remained inadequate by RMSEA (0.103). An unrestricted bifactor model fit very well (RMSEA = 0.047), but its positive-specific factor collapsed and was not interpreted. The more parsimonious S-1 representation retained a general AI-attitude factor plus a negative-wording-specific factor and showed CFI = 0.997, TLI = 0.996, RMSEA = 0.083, and SRMR = 0.045. RMSEA therefore remained borderline, whereas comparative and residual indices were strong. The general factor accounted for 72.4% of common variance (ECV = 0.724), with ωH = 0.841 and omega total = 0.980, supporting use of the overall score while acknowledging residual wording-specific variance.

For IMSE, the original five-factor ordinal model remained weak by RMSEA (0.106). Estimating the single theoretically justified residual association between reverse-worded items 10 and 12 improved fit to CFI = 0.998, TLI = 0.998, RMSEA = 0.069, and SRMR = 0.049. The two items nevertheless retained low loadings of approximately 0.25–0.29. At the level of the exact 22-item composite used in the primary analysis, internal consistency was high (Cronbach’s α = 0.971). Corrected item-total correlations ranged from 0.262 to 0.890; 20 of the 22 items showed correlations between 0.605 and 0.890, whereas the two weak reverse-worded items showed substantially lower corrected item-total correlations (item 10: 0.262; item 12: 0.306). Deleting either weak item increased α only modestly, to 0.975. In addition, the five dimensions of the 22-item model showed composite reliability values ranging from 0.840 to 0.982 and AVE values from 0.603 to 0.900. Taken together, these results provide direct measurement evidence for the reliability and broad construct coherence of the exact 22-item composite used in the primary analysis, while also indicating that the scale should not be interpreted as strictly unidimensional. Excluding them produced acceptable five-factor fit (RMSEA = 0.061) and a strong 20-item bifactor sensitivity model (RMSEA = 0.051; general ECV = 0.836; ωH = 0.954). Despite this improvement in psychometric fit, the 20-item specification was treated as a sensitivity model rather than as the primary operationalization because exclusion of items 10 and 12 was prompted by their performance in the present sample. Retaining the published 22-item composition for the primary analysis therefore provided a more conservative basis for hypothesis testing, while the 20-item analysis evaluated the robustness of the findings to removal of the two weak items. The improvement was reproduced in development and validation halves. For CES, the correlated two-factor model fit acceptably (CFI = 0.999, TLI = 0.998, RMSEA = 0.060, SRMR = 0.031), although the factor correlation and HTMT were both 0.881, indicating borderline separation (Table 3).

Detailed standardized loading ranges, reliability estimates, convergent-validity indices, and general-factor statistics are provided in Supplementary Table S2, while full discriminant-validity diagnostics are reported in Supplementary Table S3. In summary, the GAAIS S-1 model retained borderline approximate fit (RMSEA = 0.083) despite strong comparative and residual fit indices. Within the IMSE, items 10 and 12 showed weak loadings of approximately 0.25–0.29, and some first-order dimensions demonstrated limited discriminant validity, with HTMT values reaching 0.973 and 0.938. The two CES dimensions also showed borderline separation (factor r = 0.881; HTMT = 0.881). These findings support cautious interpretation of subscale-level effects. For the IMSE, use of the 22-item observed composite in the primary analysis was supported by its high total-score reliability, acceptable fit of the corresponding 22-item multidimensional measurement model, and adequate convergent reliability within its first-order dimensions; however, the weak performance of items 10 and 12 and the limited discriminant validity among several dimensions remain important qualifications. The 20-item bifactor results were interpreted solely as sensitivity evidence and were not used as psychometric justification for the original 22-item composite.

### 3.4. Common Method Variance

Harman’s unrotated single-factor analysis extracted six factors with eigenvalues greater than 1, and the first factor accounted for 38.78% of the total item variance. This result is reported as a diagnostic finding only and should not be interpreted as evidence that common method variance was absent or negligible. Because all substantive variables were obtained by self-report from the same respondents at the same measurement occasion, common method bias cannot be ruled out on the basis of Harman’s test.

### 3.5. Covariate-Adjusted Mediation Analysis

**Figure 1:**
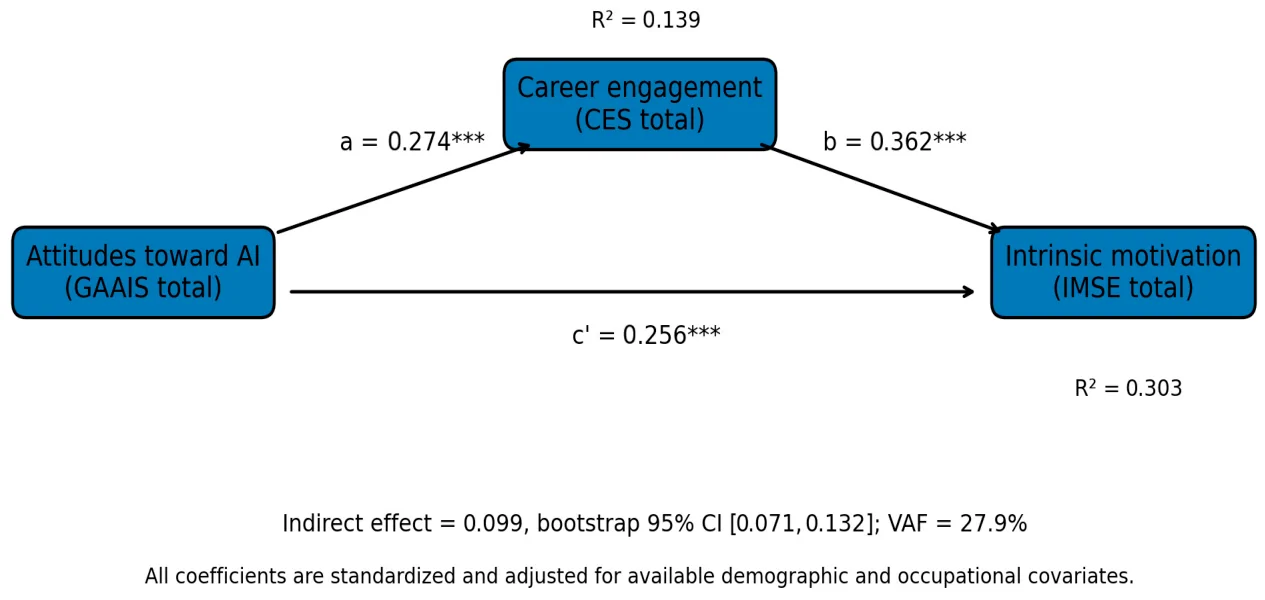
Covariate-adjusted mediation model. *** = *p* < 0.001.

The initially planned latent structural model showed inadequate overall fit and was therefore not retained for inferential interpretation: χ^2^_(24)_ = 271.61, *p* < 0.001; χ^2^/df = 11.32; CFI = 0.959; TLI = 0.939; RMSEA = 0.107, 90% CI [0.096, 0.119]; and SRMR = 0.038. As detailed in the Methods and Supplementary Table S5, the model was based on nine subscale-composite indicators and estimated 21 free parameters from 45 unique covariance-matrix elements, yielding the reported 24 degrees of freedom. Although some incremental and residual fit indices were comparatively favorable, the RMSEA and χ^2^/df indicated substantial global misfit. For this reason, the structural paths from this latent model were not interpreted as the primary hypothesis tests. As described in the Section 2, the analysis was reported as the main result using standardized observed scores in a covariate-adjusted regression mediation framework. This change was made after evaluation of the latent-model fit and therefore represents an analysis-stage modification rather than a prespecified primary approach. Because observed-score mediation does not explicitly account for measurement error, the reported coefficients represent associations among observed composite scores rather than measurement-error-corrected latent relations. More favorable AI attitudes were positively associated with career engagement, supporting H1. Career engagement and AI attitudes were each positively associated with intrinsic motivation, supporting H2 and H3. The bootstrapped indirect association through career engagement was statistically significant, consistent with H4. Because the direct association remained statistically significant, the results indicated a partial indirect-association pattern rather than evidence of a causal mediation process (Table 4 and Figure 1).

The indirect association accounted for 27.9% of the total association. The career-engagement model explained 13.9% of outcome variance (adjusted R^2^ = 0.119), and the intrinsic-motivation model explained 30.3% (adjusted R^2^ = 0.286). Incremental f^2^ values were 0.083 for AI attitudes in the career-engagement model, 0.083 for AI attitudes in the intrinsic-motivation model, and 0.162 for career engagement in the intrinsic-motivation model. Ten-fold cross-validated Q^2^ values were positive for career engagement (0.087) and intrinsic motivation (0.255). The coefficients for all demographic and occupational covariates included in the final adjusted models are reported in Supplementary Table S4. In the career-engagement model, age, education, weekly working hours, patient-services/primary-care work-unit membership, and day or mixed work schedules showed statistically significant adjusted associations. In the intrinsic-motivation model, age, gender, and shift work showed statistically significant adjusted associations. These covariate estimates are reported for transparency and were not treated as primary hypothesis tests. The 20-item IMSE sensitivity analysis produced a similar indirect association of 0.105 (95% bias-corrected bootstrap CI [0.075, 0.139]), VAF of 30.3%, intrinsic-motivation R^2^ of 0.321, and Q^2^ of 0.280. Although the 20-item specification demonstrated better psychometric fit than the 22-item specification, it was retained as a sensitivity analysis because deletion of the two weak items was informed by their performance in the present sample rather than prespecified or independently validated as a revised scale. The close correspondence of the indirect associations obtained with the 22-item and 20-item scores indicates that the principal finding did not depend on retaining the two weak reverse-worded items. Profession-stratified analyses showed a statistically significant indirect association through career engagement in both occupational strata. Among nurses/midwives (n = 266), the indirect association was 0.135 (95% bias-corrected bootstrap CI [0.083, 0.210]), whereas among the other occupational groups (n = 636), it was 0.081 (95% bias-corrected bootstrap CI [0.049, 0.121]). Interaction analyses indicated that the association between AI attitudes and career engagement was significantly stronger among nurses/midwives than among the other occupational groups (interaction β = 0.152, 95% CI [0.010, 0.294], *p* = 0.036). In contrast, occupation-group interactions were not statistically significant for the association between career engagement and intrinsic motivation (interaction β = 0.037, 95% CI [−0.127, 0.201], *p* = 0.659), the direct association between AI attitudes and intrinsic motivation (interaction β = −0.006, 95% CI [−0.186, 0.174], *p* = 0.947), or the total association between AI attitudes and intrinsic motivation (interaction β = 0.062, 95% CI [−0.125, 0.249], *p* = 0.515). Full subgroup estimates, confidence intervals, and interaction-test results are reported in Supplementary Table S1. The additional sensitivity analysis excluding all 62 students/interns (n = 840) yielded estimates that were very similar to those obtained in the full sample. The association between AI attitudes and career engagement remained statistically significant (a = 0.280, 95% CI [0.210, 0.350], *p* < 0.001), as did the association between career engagement and intrinsic motivation (b = 0.353, 95% CI [0.276, 0.429], *p* < 0.001) and the direct association between AI attitudes and intrinsic motivation (c′ = 0.263, 95% CI [0.177, 0.350], *p* < 0.001). The indirect association remained statistically significant and was virtually unchanged in magnitude (indirect association = 0.099, 95% bias-corrected bootstrap CI [0.069, 0.136], *p* < 0.001), compared with 0.099 (95% bias-corrected bootstrap CI [0.071, 0.132]) in the full sample. The total association was 0.362 (95% CI [0.272, 0.452], *p* < 0.001), and the indirect association accounted for 27.3% of the total association. These findings indicate that the principal observed association was not dependent on the inclusion of the students/interns. Full estimates for this sensitivity analysis are reported in Supplementary Table S1, Panel C.

The figure illustrates the final mediation model examining the association between attitudes toward artificial intelligence, career engagement, and intrinsic motivation among healthcare workers. Standardized path coefficients are shown after adjustment for demographic and occupational covariates. Indirect associations were estimated using 5000 nonparametric bootstrap resamples, with 95% bias-corrected (BC) bootstrap confidence intervals. All reported coefficients were statistically significant (*** *p* < 0.001).

## 4. Discussion

This study examined the relationships between attitudes toward AI, career engagement, and intrinsic motivation among healthcare workers, with particular attention to the mediating role of career engagement. The findings suggest that workforce responses to AI are shaped not only by technology-related perceptions but also by career-related processes. In this respect, the study contributes to the growing literature on the social consequences of digital transformation in healthcare.

Regarding the gender variable, refs. [49,50] found that male healthcare personnel’s positive attitudes toward AI were higher compared to female healthcare personnel. Although women constituted the majority of the sample, participants reported moderately favorable attitudes toward AI. This finding may partly reflect the relatively high educational level of the sample; however, the present data do not permit conclusions regarding the underlying reasons for this pattern. Indeed, the same studies revealed that participants with higher levels of knowledge and education had significantly higher positive attitudes toward AI compared to others.

A review addressing the effects of AI on healthcare workers’ job satisfaction and performance demonstrated that AI applications positively affect employees’ professional satisfaction and quality of work through optimizing workflows, improving diagnostic accuracy, and providing clinical decision support; however, it was emphasized that this effect largely depends on institutional support mechanisms, training infrastructure, and employees’ attitudes toward technology [51]. Consistent with this perspective, positive beliefs about AI, trust in AI, and facilitating organizational conditions were found to significantly predict nurses’ intention to integrate AI into clinical practice, emphasizing the critical role of workforce attitudes and organizational readiness in successful AI adoption [52]. Similarly, positive workforce perceptions were associated with greater acceptance of AI among allied health professionals, highlighting the importance of healthcare workers’ professional perceptions in facilitating AI adoption [53]. 

Further evidence comes from a study conducted with 357 physicians and nurses in Sweden, in which healthcare workers’ adoption attitudes toward AI were examined within the framework of paradox theory. The authors found that learning, performance, and belonging tensions positively influenced AI adoption intentions, suggesting that AI adoption is not merely a matter of technology acceptance but a multilayered process intertwined with professional identity, organizational engagement, and the individual search for meaning [54]. These results support the positive attitude finding obtained in the present study and once again confirm that attitudes toward AI cannot be evaluated independently of individual and organizational dynamics.

A study conducted on 280 healthcare workers in China found that AI applications positively affected basic psychological needs such as autonomy, competence, and relatedness, thereby enhancing workplace well-being [55]. This finding suggests that the high intrinsic motivation level identified in the present study may be associated with positive attitudes toward AI. A study conducted with physicians and nurses in an intensive care unit also stated that AI use increased autonomy levels, thereby reducing stress, enhancing motivation and performance, supporting skill variety and competence development, and enabling clinicians to focus more on patient care by relieving them of administrative burden [56]. These findings demonstrate that intrinsic motivation is not merely an individual characteristic but is also nourished by technological arrangements in the work environment, supporting the main argument of this study.

A study conducted with 97 nurses working at an educational and research hospital in the Philippines revealed that individuals with high career engagement also had stronger learning desire, eagerness to acquire new knowledge, and self-improvement orientation [57]. This finding theoretically supports the connection between the high career engagement level identified in the present study and intrinsic motivation. A study conducted with 202 employees operating in different sectors found that AI strengthened career engagement through its contribution to job arrangement processes [58]. 

These findings suggest that favorable attitudes toward AI are positively associated with career engagement, and that career engagement is, in turn, associated with intrinsic motivation—consistent with an indirect association between AI attitudes and intrinsic motivation through career engagement. Given the cross-sectional design, this pattern should not be interpreted as evidence of an underlying psychological mechanism. 

The partial indirect-association pattern involving career engagement represents the principal statistical finding of this study. The statistically significant direct and indirect associations indicate that the observed association between AI attitudes and intrinsic motivation was only partly accounted for statistically by career engagement. As the data are cross-sectional, this pattern should be interpreted as an indirect statistical association rather than a temporal or causal pathway. Alternative explanatory models remain equally plausible; for instance, higher career engagement could itself foster more favorable attitudes toward AI, or the two constructs could influence one another reciprocally over time. The present cross-sectional design does not allow us to adjudicate between these possibilities. The profession-stratified findings further indicated that the indirect association was present in both nurses/midwives and other occupational groups. However, the association between favorable AI attitudes and career engagement was significantly stronger among nurses/midwives, suggesting some occupational heterogeneity in how AI-related attitudes are linked to career-oriented engagement. Because the remaining occupation-by-predictor interactions were not statistically significant, this finding should be interpreted as path-specific heterogeneity rather than evidence that the overall association pattern differs fundamentally across occupational groups. The additional analysis excluding students/interns also produced an essentially unchanged indirect association, providing evidence that the principal finding was not driven by this training-stage subgroup. This sensitivity result supports robustness with respect to their inclusion, while not eliminating the broader limitations associated with occupational heterogeneity and non-probability sampling.

The findings should also be interpreted in light of the measurement limitations identified in the ordinal analyses. In particular, the borderline RMSEA of the GAAIS S-1 model, the two weakly loading IMSE items, the limited discriminant validity among several IMSE dimensions, and the borderline separation of the CES dimensions reduce confidence in fine-grained subscale-level interpretations. For this reason, the present study emphasizes associations among overall construct scores rather than distinct effects of individual subdimensions. Although the 20-item IMSE specification showed superior fit in the present sample, it was not adopted as the primary operationalization because its composition resulted from sample-informed deletion of two weak items and has not been established here as an independently validated replacement for the published 22-item instrument. Importantly, the indirect association remained similar when the two weak IMSE items were excluded in the 20-item sensitivity analysis, which supports the robustness of the principal observed-score finding but does not eliminate the underlying measurement uncertainty. The use of observed composite scores in the primary mediation analysis should also be considered when interpreting the magnitude of the reported associations. Because the latent structural model was not retained and measurement error was therefore no longer modeled explicitly, the estimated associations may be attenuated or otherwise affected by unreliability in the observed scores. The present results should consequently be interpreted as observed-score associations rather than as latent, measurement-error-corrected relations. This distinction is particularly relevant given the measurement limitations identified for several IMSE and CES dimensions. 

From a health system perspective, these findings suggest that workforce motivation and career engagement may be relevant not only as individual outcomes but also as factors plausibly linked to system performance, consistent with prior research reporting positive associations between employee motivation and service quality, organizational efficiency, and patient outcomes; however, system-level performance was not measured in the present study, and this link should be regarded as a theoretical possibility rather than an empirical finding. 

To the best of our knowledge, this is among the first studies to examine AI attitudes, career engagement, and intrinsic motivation simultaneously among healthcare workers using a theory-informed indirect-association framework. Therefore, the study contributes to both theoretical and applied literature by addressing an important gap in workforce-related AI research.

From a broader public health and socio-technical perspective, these findings suggest that AI integration in healthcare should not be understood solely as a technological change. Rather, workforce motivation, professional engagement, and career-related resources appear to shape how healthcare workers adapt to AI-driven transformation. 

In this regard, career engagement may plausibly shape how employees come to view technological change—for instance, as an opportunity for professional development—thereby potentially supporting workforce well-being and sustainable health system transformation; however, this interpretive process was not directly measured in the present study and should be treated as a theoretical possibility rather than an empirical finding.

These findings may be particularly relevant for developing and emerging health systems, where resource constraints and workforce challenges are more pronounced. In such contexts. Workforce readiness and motivation may be relevant considerations in discussions of AI-related organizational change; however, the present study did not evaluate AI implementation outcomes or integration success. The use of observed total scores should also be considered when interpreting the magnitude of the reported associations. Because this approach does not explicitly model measurement error, the estimates represent associations among observed composite scores rather than latent, measurement-error-corrected relations.

## 5. Limitations and Future Research

This study has several limitations. First, its cross-sectional design limits causal interpretation. Second, the data were based on self-reports, which may introduce common bias and social desirability effects. Third, the use of non-probability snowball sampling limits the representativeness and generalizability of the findings. Because recruitment was conducted through online professional networks, no population-based sampling frame was available, individual inclusion probabilities could not be determined, and a conventional response rate could not be calculated. This recruitment strategy may also have introduced self-selection bias, as healthcare workers with greater interest in AI or digital technologies may have been more likely to participate, potentially resulting in more favorable attitudes toward AI in the analytic sample. Conversely, healthcare workers with limited internet access, lower digital engagement, or less interest in technological change may have been underrepresented. Accordingly, the observed distribution of AI attitudes should not be interpreted as nationally representative of all healthcare workers in Türkiye, and the generalizability of both the descriptive findings and the observed associations should be considered with caution. Future studies using probability-based or institutionally stratified sampling strategies are needed to assess the robustness of these findings across the broader healthcare workforce. In addition, although the questionnaire-level screening criteria are now explicitly described, reason-specific counts were not retained for the 382 excluded submissions; therefore, the relative contribution of incompleteness, erroneous or internally inconsistent completion, and non-differentiated response patterns to the final exclusions cannot be quantified. The extended data-collection period represents an additional limitation. Data were collected over approximately 11 months, from April 2025 to February 2026, during a period in which healthcare workers’ exposure to, familiarity with, and organizational implementation of AI may have changed. Consequently, participants responding earlier and later in the data-collection period may have been exposed to different technological and organizational contexts. Because individual response dates or survey months were not retained in the analytic dataset, temporal variation could not be evaluated or adjusted for in sensitivity analyses. The possibility of calendar-time-related heterogeneity therefore cannot be excluded. Future studies examining rapidly evolving technologies such as AI should retain response timestamps and consider survey month or another indicator of calendar time as a covariate or stratification variable. Fourth, years of professional experience were not collected; because experience may be related to career engagement, motivation, and attitudes toward technological change, its absence represents a potential residual confounder that should be addressed in future research. The occupational heterogeneity of the sample should also be considered when interpreting the findings. Although profession was included as a covariate and subgroup analyses were conducted, broad professional categories cannot fully capture differences in AI exposure, clinical responsibilities, professional autonomy, career trajectories, or motivational contexts across healthcare occupations. Consequently, residual heterogeneity may remain within and between occupational groups. Future studies should examine more occupationally homogeneous samples or use sufficiently large profession-specific samples to evaluate whether the observed associations differ across distinct healthcare professions and levels of AI exposure. Finally, several measurement-related limitations warrant caution. The GAAIS S-1 model retained borderline approximate fit (RMSEA = 0.083), despite strong CFI, TLI, and SRMR values. Within the 22-item IMSE, two reverse-worded items showed weak standardized loadings of approximately 0.25–0.29, and several first-order dimensions demonstrated limited discriminant validity, including HTMT values of 0.973 and 0.938. The two CES dimensions also showed borderline separation (factor correlation = 0.881; HTMT = 0.881). These findings reduce confidence in fine-grained interpretations of the individual subdimensions and indicate that the measurement structure was not uniformly strong. The primary analyses therefore focused on overall standardized construct scores, and a 20-item IMSE sensitivity analysis was conducted to assess the influence of the two weakly loading items. For the IMSE, this decision was based on measurement evidence derived directly from the 22-item version used in the primary analysis, including high total-score internal consistency and acceptable fit of its five-factor measurement model, rather than on the general-factor indices obtained from the modified 20-item sensitivity specification. Nevertheless, the two weak items and incomplete discriminant separation indicate that the 22-item total score should be interpreted as a broad observed composite and not as evidence of a strictly unidimensional construct. A further limitation concerns the transition from the initially planned latent structural model to observed-score mediation after evaluation of model fit. Although this decision avoided interpreting structural relations within a poorly fitting latent model and was consistent with the study’s focus on overall construct scores, observed-score regression does not explicitly model measurement error. As a result, unreliability in the composite scores may attenuate regression coefficients and indirect associations, while non-random measurement error may introduce additional bias. Neither standardization, heteroskedasticity-robust standard errors, nor bootstrap confidence intervals corrects for this source of uncertainty. Accordingly, the observed-score estimates should not be interpreted as equivalent to latent-variable estimates or as measurement-error-corrected effects. Future studies should independently replicate the measurement structures and evaluate the hypothesized associations within well-fitting latent-variable models specified independently of the observed structural results. The similar indirect association obtained in this sensitivity analysis supports the robustness of the principal observed-score result; however, it does not remove the underlying measurement limitations. Conversely, the improved fit of the 20-item specification should not be interpreted as establishing a revised version of the IMSE, because the item exclusions were data-informed within the present sample and require independent replication. Future research should independently replicate the factor structures, further examine the performance of reverse-worded IMSE items, and evaluate the discriminant validity of the IMSE and CES dimensions in other healthcare samples before strong subscale-specific conclusions are drawn.

In addition, common method variance represents a potential limitation because all substantive variables were measured using self-report instruments obtained from the same respondents at a single measurement occasion. Harman’s single-factor test indicated that the first unrotated factor accounted for 38.78% of total variance; however, this diagnostic result does not establish that common method variance was absent or negligible. Given the same-source, same-time self-report design, common method bias remains a plausible source of shared variance. Future studies should incorporate multi-source, temporally separated, or longitudinal measurements and, where feasible, more rigorous statistical approaches to evaluate method-related variance.

## 6. Conclusions

This study examined the relationships between attitudes toward artificial intelligence, career engagement, and intrinsic motivation among healthcare workers using a cross-sectional indirect-association framework. More favorable attitudes toward AI were positively associated with both career engagement and intrinsic motivation. A statistically significant indirect association between AI attitudes and intrinsic motivation was also observed through career engagement. Given the cross-sectional design, this pattern should be interpreted as an indirect statistical association rather than evidence of a temporal or causal mediation mechanism.

From a public health and health systems perspective, these findings suggest that workforce-related factors, such as attitudes toward AI, career engagement, and intrinsic motivation, may be relevant alongside technological readiness in shaping how healthcare workers adapt to AI-related change. As this study did not directly measure AI implementation outcomes or the success of AI integration, this link should be regarded as a plausible extension of our findings rather than an empirical conclusion. Although these variables were not experimentally manipulated in this study, it is plausible that supporting healthcare workers through career development opportunities, AI-related training, and motivational strategies could strengthen adaptation to digital transformation; this remains a practical implication to be tested in future intervention research rather than a conclusion supported directly by the present findings. It should also be noted that this study assessed healthcare workers’ attitudes toward AI rather than their actual use of AI systems or the extent to which they worked in AI-enabled clinical settings; these represent distinct constructs that were not measured here. Future studies may benefit from longitudinal and comparative designs to further examine these relationships across different healthcare contexts.

## Figures and Tables

**Table 1:** Demographic and occupational characteristics of participants (N = 902).

Variable	Category	n	%
Age	≤25	379	42.0
	26–35	221	24.5
	36–45	158	17.5
	≥46	144	16.0
Gender	Women	599	66.4
	Men	303	33.6
Marital status	Married	370	41.0
	Single	532	59.0
Education	Secondary	56	6.2
	Associate	163	18.1
	Bachelor’s	561	62.2
	Master’s	82	9.1
	Doctorate	40	4.4
Profession	Physicians	181	20.1
	Nurses and midwives	266	29.5
	Health technicians	165	18.3
	Other health professionals	155	17.2
	Managers/specialists	20	2.2
	Administrative/support	53	5.9
	Students/interns	62	6.9
Work schedule	On-call	189	21.0
	Day	462	51.2
	Shift	39	4.3
	Mixed	212	23.5
Weekly hours	<40	180	20.0
	40	370	41.0
	>40	352	39.0
Work unit	Clinical and inpatient wards	288	31.9
	Emergency and intensive care units	103	11.4
	Operating room, delivery room, and sterilization	68	7.5
	Administrative and management units	114	12.6
	Diagnostic, support, and technical units	130	14.4
	Patient services and primary care	64	7.1
	Education and other	135	15.0

**Table 2:** Descriptive statistics, reliability, and correlations among focal variables.

Variable	M	SD	α	1	2	3
1. Attitudes toward AI	3.087	0.879	0.959	—	0.298	0.359
2. Career engagement	3.540	0.688	0.936	0.298	—	0.429
3. Intrinsic motivation	3.767	0.781	0.971	0.359	0.429	—

Note. All correlations are statistically significant at *p* < 0.001. Scores range from 1 to 5. α = Cronbach’s alpha.

**Table 3:** Fit of selected ordinal measurement models.

Scale	Model	DWLS χ^2^	df	CFI	TLI	RMSEA (90% CI)	SRMR
GAAIS	Bifactor S-1 (general + negative-specific)	1156.62	162	0.997	0.996	0.083 [0.078, 0.087]	0.045
IMSE	22-item five-factor + reverse-wording residual	1057.03	198	0.998	0.998	0.069 [0.065, 0.074]	0.049
IMSE	20-item five-factor sensitivity	700.21	160	0.999	0.999	0.061 [0.057, 0.066]	0.036
IMSE	20-item bifactor sensitivity	507.18	150	0.999	0.999	0.051 [0.047, 0.056]	0.030
CES	Two correlated factors	109.41	26	0.999	0.998	0.060 [0.048, 0.071]	0.031

Note. Estimates are based on polychoric correlations and DWLS. The 20-item IMSE models are sensitivity analyses and are not presented as a newly validated replacement scale. Items 10 and 12 were excluded only after their weak loadings were identified in the present sample; therefore, the published 22-item composition was retained as the primary operationalization to avoid basing the primary hypothesis test on sample-informed item deletion.

**Table 4:** Covariate-adjusted direct, indirect, and total associations.

Path	Standardized Estimate	95% CI	*p*	Result
AI attitudes → Career engagement (a)	0.274	[0.206, 0.342]	<0.001	H1 consistent
Career engagement → Intrinsic motivation (b)	0.362	[0.293, 0.432]	<0.001	H2 consistent
AI attitudes → Intrinsic motivation (c′)	0.256	[0.177, 0.340]	<0.001	H3 consistent
Indirect: AI attitudes → Career engagement → Intrinsic motivation	0.099	[0.071, 0.132]	<0.001	H4 consistent
Total association (c)	0.356	[0.272, 0.441]	<0.001	Partial indirect-association pattern

Note. The 95% confidence interval for the indirect association was obtained from 5000 nonparametric bootstrap resamples using the bias-corrected (BC) method. All models adjusted for age, gender, marital status, education, profession, work unit, work schedule, and weekly working hours. Years of experience was unavailable.

## Data Availability

Due to ethical restrictions and participant privacy considerations, the raw data are not publicly available. However, a minimal anonymized dataset sufficient to reproduce the principal analyses, subject to applicable ethical and privacy requirements, is available from the corresponding author upon reasonable request.

## Supplementary Materials

The following supporting information can be downloaded at: https://www.mdpi.com/article/10.3390/healthcare14183078/s1, Table S1, profession-stratified mediation estimates and occupation-by-predictor interaction tests, including Panel A: Profession-stratified mediation analyses, Panel B: Occupation-by-predictor interaction tests, and Panel C: Sensitivity analysis excluding students/interns (n = 840); Table S2, loading ranges, reliability, convergent validity, and general-factor evidence; Table S3, selected discriminant-validity diagnostics; Table S4, covariate coefficients from the final adjusted regression models; Table S5, specification and degrees-of-freedom accounting for the initially planned latent structural model; Supplementary Code, Python scripts used for data processing and the principal statistical analyses.
