# Supplementary material for: Attitudes Toward Artificial Intelligence, Career Engagement, and Intrinsic Motivation Among Healthcare Workers in Türkiye: A Cross-Sectional Study of Indirect Associations
Source: Healthcare (Basel). 2026 Sep 18;14(18):3078. doi: 10.3390/healthcare14183078 (PMC13606287; doi:10.3390/healthcare14183078)
Supplement: Supplementary file 1 [file healthcare-14-03078-s001.zip › Table S1.pdf]

Table S1. Profession-stratified mediation estimates and occupation-by-predictor interaction tests

Panel A. Profession-stratified mediation analyses

**Occupational group**	**Path**	**Standardized estimate**	**95% CI**	**p**
Nurses/midwives (n=266)	AI attitudes → Career engagement (a)	.378	[.258, .497]	<.001
	Career engagement → Intrinsic motivation (b)	.358	[.210, .506]	<.001
	AI attitudes → Intrinsic motivation (c′)	.250	[.102, .398]	.001
	Indirect association (a×b)	**.135**	**[.083, .210]**	<.001
	Total association (c)	.385	[.233, .537]	<.001
Other occupational groups (n=636)	AI attitudes → Career engagement (a)	.229	[.144, .314]	<.001
	Career engagement → Intrinsic motivation (b)	.356	[.269, .442]	<.001
	AI attitudes → Intrinsic motivation (c′)	.262	[.160, .363]	<.001
	Indirect association (a×b)	**.081**	**[.049, .121]**	<.001
	Total association (c)	.343	[.236, .450]	<.001

Panel B. Occupation-by-predictor interaction tests

**Interaction**	**βinteraction**	**95% CI**	**p**
Nurses/midwives × AI attitudes → Career engagement	**.152**	**[.010, .294]**	**.036**
Nurses/midwives × Career engagement → Intrinsic motivation	.037	[−.127, .201]	.659
Nurses/midwives × AI attitudes → Intrinsic motivation (direct c′)	−.006	[−.186, .174]	.947
Nurses/midwives × AI attitudes → Intrinsic motivation (total c)	.062	[−.125, .249]	.515

Note. All focal variables were standardized using the full-sample means and standard deviations. Regression-path estimates and confidence intervals were based on HC3 heteroskedasticity-consistent standard errors. Confidence intervals for indirect associations were obtained from 5,000 nonparametric bootstrap resamples using the bias-corrected (BC) method. Models were adjusted for age, gender, marital status, education, work unit, work schedule, and weekly working hours. Profession was omitted from the nurses/midwives subgroup model because it was invariant within that subgroup and was retained as a categorical covariate in the other-occupational-groups model. Interaction models retained the profession indicators used in the primary analysis. Positive interaction coefficients indicate a stronger association among nurses/midwives relative to the other occupational groups.

Panel C. Sensitivity analysis excluding students/interns (n = 840)

Path	Standardized estimate	95% CI	p
AI attitudes → Career engagement (a)	.280	[.210, .350]	<.001
Career engagement → Intrinsic motivation (b)	.353	[.276, .429]	<.001
AI attitudes → Intrinsic motivation (c′)	.263	[.177, .350]	<.001
Indirect association (a×b)	.099	[.069, .136]	<.001
Total association (c)	.362	[.272, .452]	<.001

Note. This sensitivity analysis excluded all 62 participants classified as students/interns, leaving n = 840. Focal scores were restandardized within the restricted sample. The same covariate structure as in the primary analysis was retained, except that the students/interns profession category was no longer present. HC3 heteroskedasticity-consistent standard errors were used for regression paths. The confidence interval for the indirect association was obtained from 5,000 nonparametric bootstrap resamples using the bias-corrected (BC) method. Career-engagement $R^2 = .145$ (adjusted $R^2 = .124$); intrinsic-motivation $R^2 = .297$ (adjusted $R^2 = .279$); VAF = 27.3%.

The career-engagement and intrinsic-motivation models explained 25.5% (adjusted $R^2 = .210$) and 36.3% (adjusted $R^2 = .322$) of the variance, respectively, among nurses/midwives, compared with 11.3% (adjusted $R^2 = .085$) and 29.7% (adjusted $R^2 = .273$) among the other occupational groups.
