# Supplementary material for: Attitudes Toward Artificial Intelligence, Career Engagement, and Intrinsic Motivation Among Healthcare Workers in Türkiye: A Cross-Sectional Study of Indirect Associations
Source: Healthcare (Basel). 2026 Sep 18;14(18):3078. doi: 10.3390/healthcare14183078 (PMC13606287; doi:10.3390/healthcare14183078)
Supplement: Supplementary file 1 [file healthcare-14-03078-s001.zip › Table S2.pdf]

Table S2. Loading ranges, reliability, convergent validity, and general-factor evidence.

Scale	Selected model	Factor	Items	Loading range	CR	AVE	Additional evidence
GAAIS	S-1 bifactor	General AI attitude	20	.348-.940	—	—	ECV=.724; ωH=.841; ωtotal=.980
GAAIS	S-1 bifactor	Negative-specific	8	.590-.807	—	—	Residual wording/domain variance
IMSE	22-item primary composite	Overall observed score	22	—	—	—	α=.971; corrected item-total r=.262–.890; 20/22 items=.605–.890; item 10=.262; item 12=.306; α if item 10 deleted=.975; α if item 12 deleted=.975
IMSE	22-item + wording residual	Utility	6	.936-.963	.982	.900	
IMSE	22-item + wording residual	Belief	4	.249-.995	.840	.603	Item 10 weak
IMSE	22-item + wording residual	Perceived competence	5	.260-.966	.911	.695	Item 12 weak
IMSE	22-item + wording residual	Negative affect	4	.783-.976	.955	.842	
IMSE	22-item + wording residual	Interest/enjoyment	3	.939-.958	.964	.899	
CES	Two correlated factors	Planning/evaluation	5	.792-.936	.937	.750	
CES	Two correlated factors	Research/networkin g	4	.836-.908	.924	.753	Factor r=.881; HTMT=.881

Note. *CR = composite reliability; AVE = average variance extracted; ECV = explained common variance; ωH = omega hierarchical. GAAIS positive items define the S-1 reference domain and therefore have no positive-specific factor.*

The Fornell-Larcker and HTMT results were not uniformly favorable. The two GAAIS first-order domains were adequately differentiated. The total-score reliability diagnostics reported for the IMSE refer specifically to the exact 22-item composite used in the primary analysis. The 20-item bifactor indices apply only to the sensitivity specification and should not be interpreted as evidence for the dimensionality or validity of the original 22-item composite. The 22-item composite is therefore interpreted as a broad observed measure of intrinsic motivation rather than as a strictly unidimensional latent score. In contrast, several IMSE factor pairs exceeded conventional HTMT thresholds and failed the Fornell-Larcker comparison, while the two CES dimensions were borderline. These findings support cautious use of subdimensions and favor overall-score interpretation for the primary indirect-association analysis.
