# Supplementary material for: Attitudes Toward Artificial Intelligence, Career Engagement, and Intrinsic Motivation Among Healthcare Workers in Türkiye: A Cross-Sectional Study of Indirect Associations
Source: Healthcare (Basel). 2026 Sep 18;14(18):3078. doi: 10.3390/healthcare14183078 (PMC13606287; doi:10.3390/healthcare14183078)
Supplement: Supplementary file 1 [file healthcare-14-03078-s001.zip › Table S3.pdf]

Table S3. Selected discriminant-validity diagnostics.

Factor pair	Factor r	HTMT	√AVE values	Interpretation
GAAIS positive vs reversed-negative	.544	.537	.885 / .854	Fornell-Larcker and HTMT supported
IMSE perceived competence vs interest/enjoyment	.962	.973	.836 / .948	Discriminant validity not supported
IMSE perceived competence vs negative affect	.915	.938	.836 / .917	Discriminant validity limited
CES planning vs research/networking	.881	.881	.866 / .868	Borderline; correlation exceeds both diagonals

Taken together, these findings indicate that the measurement structure should not be interpreted as uniformly strong across all scales and subdimensions. The GAAIS S-1 model retained borderline approximate fit according to RMSEA despite strong comparative and residual fit indices. For the IMSE, two reverse-worded items showed weak loadings, and the high correlations and HTMT values among several first-order factors indicated limited discriminant validity. Similarly, the two CES dimensions showed only borderline empirical separation. Accordingly, the primary hypothesis tests were based on standardized overall scores rather than on distinct subscale-level effects, and no substantive mediation inference was based on the assumption that the IMSE or CES subdimensions were fully discriminant. The 20-item IMSE sensitivity analysis was additionally used to evaluate whether the principal indirect association depended on the two weakly loading items.
