# Supplementary material for: Attitudes Toward Artificial Intelligence, Career Engagement, and Intrinsic Motivation Among Healthcare Workers in Türkiye: A Cross-Sectional Study of Indirect Associations
Source: Healthcare (Basel). 2026 Sep 18;14(18):3078. doi: 10.3390/healthcare14183078 (PMC13606287; doi:10.3390/healthcare14183078)
Supplement: Supplementary file 1 [file healthcare-14-03078-s001.zip › Table S4.pdf]

Table S4. Covariate coefficients from the final adjusted regression models.

Covariate / contrast	Career engagement model B [95% CI], p	Intrinsic motivation model B [95% CI], p
Age, one-category increase	−.132 [−.217, −.046], .003	.078 [.005, .152], .037
Men vs women	−.136 [−.283, .010], .068	−.249 [−.382, −.115], <.001
Single vs married	−.140 [−.321, .041], .130	−.131 [−.292, .030], .111
Education, one-category increase	.105 [.013, .196], .026	.012 [−.075, .098], .792
Nurses/midwives vs physicians	−.090 [−.286, .106], .367	−.056 [−.232, .120], .533
Health technicians vs physicians	−.092 [−.332, .147], .449	.101 [−.108, .309], .344
Other health professionals vs physicians	−.046 [−.282, .191], .704	−.104 [−.302, .094], .301
Managers/specialists vs physicians	.283 [−.085, .652], .132	−.213 [−.656, .230], .346
Administrative/support vs physicians	.002 [−.390, .393], .993	.158 [−.243, .559], .440
Students/interns vs physicians	−.096 [−.397, .205], .532	.022 [−.241, .284], .871
Emergency/intensive care vs clinical/inpatient wards	.217 [−.001, .436], .051	.052 [−.143, .248], .599
OR/delivery/sterilization vs clinical/inpatient wards	−.125 [−.393, .143], .361	.099 [−.119, .317], .374
Administrative/management vs clinical/inpatient wards	.051 [−.180, .281], .666	.046 [−.155, .246], .656
Diagnostic/support/technical vs clinical/inpatient wards	.035 [−.196, .266], .765	−.133 [−.335, .068], .195
Patient services/primary care vs clinical/inpatient wards	−.286 [−.558, −.014], .040	.179 [−.052, .409], .129
Education/other vs clinical/inpatient wards	−.116 [−.330, .099], .290	−.145 [−.357, .067], .180
Day vs on-call	.233 [.039, .427], .019	.154 [−.014, .322], .072
Shift vs on-call	−.076 [−.480, .328], .712	.338 [.002, .674], .049
Mixed vs on-call	.210 [.002, .418], .048	.067 [−.116, .250], .473

Covariate / contrast	Career engagement model B [95% CI], p	Intrinsic motivation model B [95% CI], p
Weekly hours, one-category increase	.132 [.036, .228], .007	−.014 [−.106, .078], .760

Note. B values are regression coefficients from models with standardized outcome scores; therefore, coefficients are expressed in outcome standard-deviation units. The focal AI-attitude, career-engagement, and intrinsic-motivation scores were standardized before analysis. HC3 heteroskedasticity-consistent standard errors were used. Age, education, and weekly working hours were entered as ordered covariates; their coefficients represent a one-category increase. Binary and dummy-coded categorical coefficients represent contrasts with the reference categories specified in Section 2.4. Reference categories were women for gender, married for marital status, physicians for profession, clinical and inpatient wards for work unit, and on-call for work schedule.
