# Supplementary material for: Attitudes Toward Artificial Intelligence, Career Engagement, and Intrinsic Motivation Among Healthcare Workers in Türkiye: A Cross-Sectional Study of Indirect Associations
Source: Healthcare (Basel). 2026 Sep 18;14(18):3078. doi: 10.3390/healthcare14183078 (PMC13606287; doi:10.3390/healthcare14183078)
Supplement: Supplementary file 1 [file healthcare-14-03078-s001.zip › Table S5.pdf]

Table S5. Specification and degrees-of-freedom accounting for the initially planned latent structural model.

Component	Specification
**Latent factor: AI attitudes**	Indicators: positive-attitude GAAIS subscale (PGAAIS) and negative-attitude GAAIS subscale (NGAAIS). PGAAIS loading fixed to 1.0; NGAAIS loading freely estimated.
**Latent factor: Career engagement**	Indicators: Planning/Evaluation (PLEV) and Research/Networking (RENE). PLEV loading fixed to 1.0; RENE loading freely estimated.
**Latent factor: Intrinsic motivation**	Indicators: Utility, Belief, Perceived Competence, Negative Affect, and Interest/Enjoyment. Utility loading fixed to 1.0; remaining four loadings freely estimated.
**Structural paths**	AI attitudes → Career engagement; Career engagement → Intrinsic motivation; AI attitudes → Intrinsic motivation. All three paths freely estimated.
**Indicator residuals**	Nine residual variances freely estimated. Cross-loadings and correlated indicator residuals fixed to zero.
**Latent variances/disturbances**	Variance of exogenous AI-attitude factor and residual variances of career engagement and intrinsic motivation freely estimated. No covariance specified between endogenous disturbances.
**Observed covariance moments**	9(9+1)/2 = 45
**Free factor loadings**	6
**Indicator residual variances**	9
**Structural paths**	3
**Latent variance/disturbance parameters**	3
**Total free parameters**	21
**Degrees of freedom**	**45 – 21 = 24**
**Model fit**	$\chi^2(24)=271.61$, $p<.001$; $\chi^2/df=11.32$; CFI=.959; TLI=.939; RMSEA=.107, 90% CI [.096,.119]; SRMR=.038

Note. The model was specified using nine observed subscale-composite indicators rather than individual items. One marker loading per latent construct was fixed to 1.0 for identification. Structural path estimates from this model were not interpreted because the model was rejected on the basis of inadequate global fit.
